# Diagnostic Value of IgA Antibody Measurement in Tick-Borne Spotted Fever (Astrakhan Rickettsial Fever)

**DOI:** 10.1128/spectrum.01687-21

**Published:** 2022-04-25

**Authors:** Nina S. Smirnova, Alexey V. Kostarnoy, Alexey V. Kondratev, Petya G. Gancheva, Daniil A. Grumov, Alexander L. Gintsburg

**Affiliations:** a Laboratory of Ecology of Rickettsia, N. F. Gamaleya National Research Center of Epidemiology and Microbiology, Moscow, Russia; b Laboratory of Immunobiotechnology, N. F. Gamaleya National Research Center of Epidemiology and Microbiology, Moscow, Russia; c Laboratory of Gene Engineering of Pathogenic Microorganisms, N. F. Gamaleya National Research Center of Epidemiology and Microbiology, Moscow, Russia; Quest Diagnostics Nichols Institute

**Keywords:** ELISA, IgA, rickettsiosis, serology, tick-borne spotted fever

## Abstract

Tick-borne spotted fevers caused by *Rickettsia* occur worldwide. The symptoms of this bacterial infection are similar to those of viral infection, and thus, diagnostic accuracy has special clinical importance. One of the commonly used methods for the diagnosis of tick-borne spotted fever is enzyme-linked immunosorbent assay (ELISA), which is based on estimation of the presence of specific IgM antibodies in blood. However, IgA analysis has not been used for the diagnosis of rickettsial diseases thus far. We investigated the diagnostic value of IgA antibody determination using patient sera collected in the Astrakhan region of Russia, where an isolated site of Astrakhan rickettsial fever (ARF) caused by Rickettsia conorii subsp. *caspia* is located. Our investigation was performed on serum samples collected from 185 patients diagnosed with Astrakhan rickettsial fever from May to October 2019. Western blot analysis revealed that specific IgA antibodies, as well as IgM antibodies, from patient sera bind to high-molecular-weight pathogen proteins with similar masses. The obtained data show that the determination of IgM alone allows for serological confirmation of diagnosis in only 46.5% of cases but that the determination of both IgM and IgA increases this rate to 66.5%. Taken together, the findings show an important diagnostic value of IgA evaluation for tick-borne spotted fever rickettsiosis.

**IMPORTANCE** Tick-borne spotted fevers caused by *Rickettsia* occur worldwide. The symptoms of this bacterial infection are similar to the symptoms of viral infection, and thus, diagnostic accuracy has special clinical importance. The most serious spotted fever group rickettsiosis is Rocky Mountain fever in the United States, which is caused by Rickettsia rickettsii, and disease complications can lead to hemiparesis, blindness, or amputation. Rickettsia conorii subsp. *caspia* causes a rickettsial spotted fever named Astrakhan rickettsial fever (ARF). One of the commonly used methods for the diagnosis of tick-borne spotted fevers is ELISA, which is based on estimation of the presence of specific IgM antibodies in blood, though IgA has not been used for the diagnosis of rickettsial diseases thus far. In this study, we showed that both IgA and IgM should be analyzed in the blood serum samples of patients to significantly enhance the accuracy of diagnostics of tick-borne spotted fever rickettsiosis.

## INTRODUCTION

The *Rickettsia* genus (order *Rickettsiales*, family *Rickettsiaceae*) consists of Gram-negative rod-like, coccoidal, or pleomorphic bacteria without flagella that are 0.3 to 0.5 μm in diameter and 0.8 to 2.0 μm in length (1). The cell wall of these bacteria contains muramic and glucuronic acids, lipopolysaccharides (LPSs), lipoproteins, and proteins, including the surface immunogenic proteins OmpВ and OmpA (2). Rickettsias are obligate intracellular parasites, and bacteria grow and reproduce only in the cells of living organisms, particularly affecting reticular tissue and the vascular endothelium (1, 3, 4).

Rickettsial diseases are usually divided into two groups: the spotted fever group (SFG) and the typhus group (TG) (1, 5). Seventeen *Rickettsia* species from the SFG are pathogenic. The most serious spotted fever group rickettsiosis is Rocky Mountain fever, which occurs in the United States and is caused by Rickettsia rickettsii. Rocky Mountain fever complications can lead to hemiparesis, blindness, or amputation following gangrene (6). Tick-borne spotted fevers caused by *Rickettsia* are found worldwide and include diseases such as Pacific Coast tick fever, caused by Rickettsia philipii; rickettsialpox, caused by Rickettsia akari; Rickettsia parkeri rickettsiosis; Mediterranean spotted fever (MSF), caused by Rickettsia conorii; and North Asian tick typhus, caused by Rickettsia sibirica. Rickettsia conorii subsp. *caspia*, a causative agent of Astrakhan rickettsial fever (ARF), which is an SFG member, plays an important role in the epidemiology of rickettsial diseases in the Russian Federation (7, 8).

The site of ARF is unique because the disease spreads strictly within the Astrakhan region of Russia. The tick Rhipicephalus sanguineus, which feeds on and is carried by dogs (9), is considered the vector for Rickettsia conorii subsp. *caspia*.

A variety of methods are used for the diagnosis of spotted fever group rickettsiosis, such as the Weil Felix test, immunofluorescence antibody assay (IFA), complement fixation assay, and Western blotting (5, 10). However, spotted fever group rickettsiosis is routinely diagnosed by pathogen detection via PCR or by evaluation of specific IgM or rising IgG serum antibody titers using enzyme-linked immunosorbent assay (ELISA) (11).

The symptoms of rickettsial fevers are similar to those of viral infections (12). Thus, the accuracy of diagnostic methods is crucial for choosing the appropriate treatment strategy. Here, we reveal that evaluation of specific IgA antibodies in addition to IgM antibodies for ARF is important because it significantly increases diagnostic accuracy.

## RESULTS

**FIG 1:**
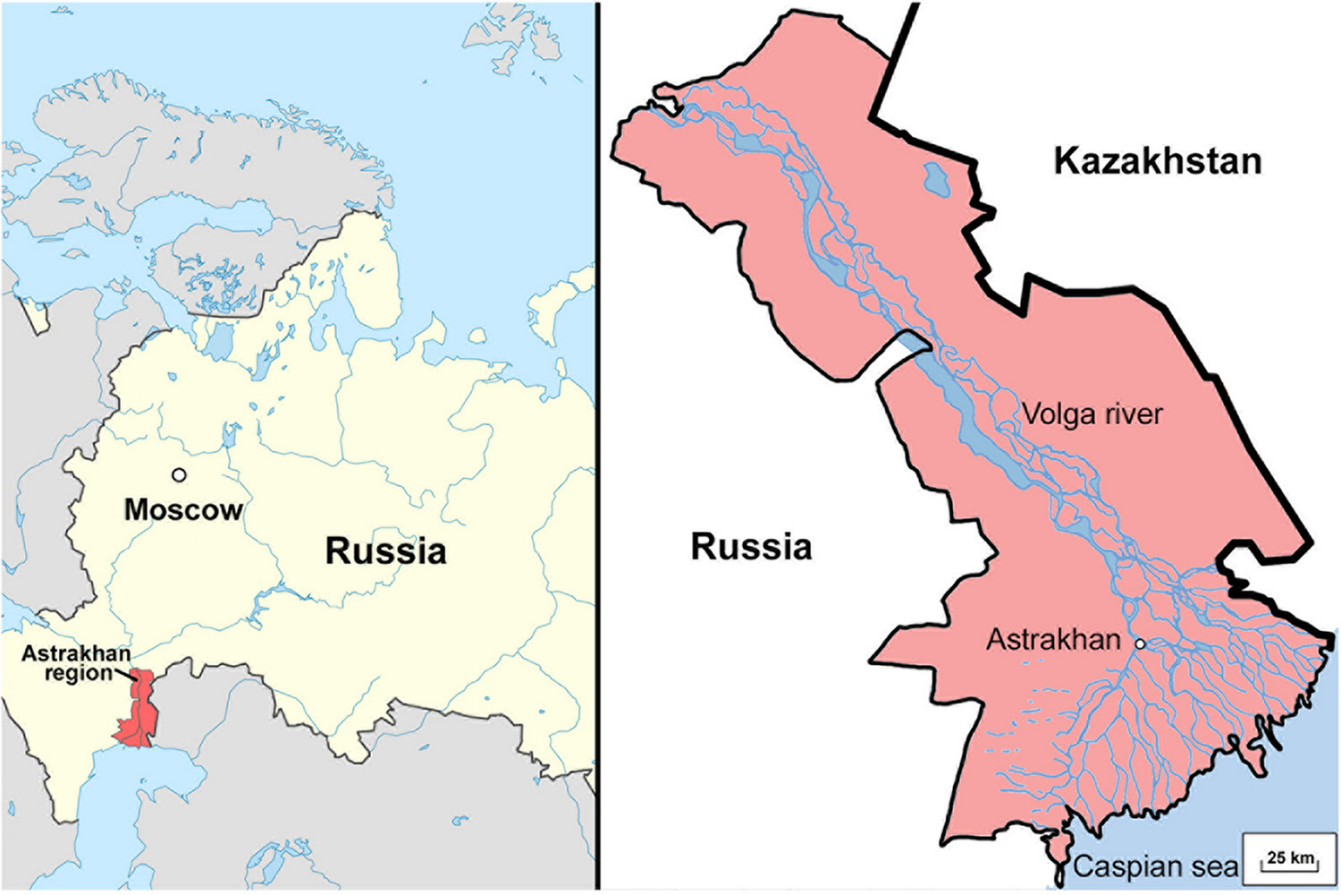
Map showing the location of the Astrakhan region, where ARF is endemic. Map drawn by author A. V. Kondratev.

In this study, we used blood serum from patients with Astrakhan rickettsial fever. The disease is endemic to the Astrakhan region of Russia. The Astrakhan region is densely populated and located in southern Russia in the lower Volga River basin, where the river flows into the Caspian Sea, forming the largest river delta in Europe, with marshes and lakes (Fig. 1).

In total, 185 samples of serum from 185 patients with ARF (97 men and 83 women aged 1 to 88 years, mean of 45.0 ± 23.6 years) were evaluated. Blood for analysis was taken at various disease stages (days 1 to 16 after infection onset) from May to October 2019. We also include in the study two control groups of sera: (i) sera from Q-fever-seropositive patients, whose seropositivity was determined using a commercially available assay (Coxiella burnetii ELISA kits; Vircell, Spain), and (ii) serum from healthy persons who were seronegative for ARF IgG/IgM and Q fever IgG/IgM, with the fact being determined via commercially available assays from Vircell, Spain.

**FIG 2:**
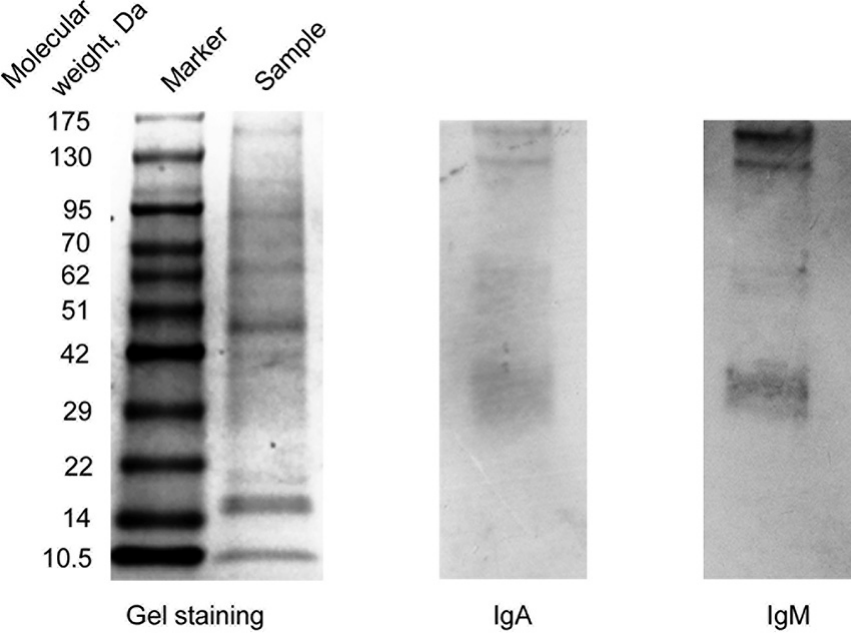
Western blotting of the combined serum specimens of patients with ARF. Rickettsia conorii proteins were separated via SDS-PAGE and stained using EZblue reagent (left panel) or transferred onto nitrocellulose membranes and probed with the combined patient serum samples, followed by incubation with IgA- or IgM-specific secondary antibodies (right panels).

In the first stage, we assessed the presence of specific IgA antibodies in sera via Western blot analysis. Bacteria were sonicated, and the obtained supernatant was separated via SDS-PAGE and transferred onto nitrocellulose membranes. The membranes were probed with the combined patient serum samples, followed by incubation with anti-IgA or anti-IgM secondary antibodies. Western blot evaluation revealed that specific IgA antibodies bind to high-molecular-weight protein bands (Fig. 2). IgA and IgM antibodies recognized proteins with very similar masses.

Then, the presence of specific IgM and IgA to Rickettsia conorii subsp. *caspia* in patient sera was qualitatively evaluated by ELISA. A designed and validated N. F. Gamaleya National Research Center for Epidemiology and Microbiology ELISA kit was used for analysis. To obtain an antigen substance, lyophilized bacteria were resuspended, sonicated, and then purified via liquid chromatography from egg proteins. A representative chromatogram of the purification process is presented in Fig. S1 in the supplemental material. Each fraction was diluted 1:20 and then tested for antigenic activity via ELISA using 10 combined sera which were seropositive in a commercially available assay (Rickettsia conorii ELISA IgG/IgM kit; Vircell, Spain). As only fractions corresponding to the first chromatography peak had antigenic activity (Fig. S1), they were used in further experiments as an antigenic substance.

**FIG 3:**
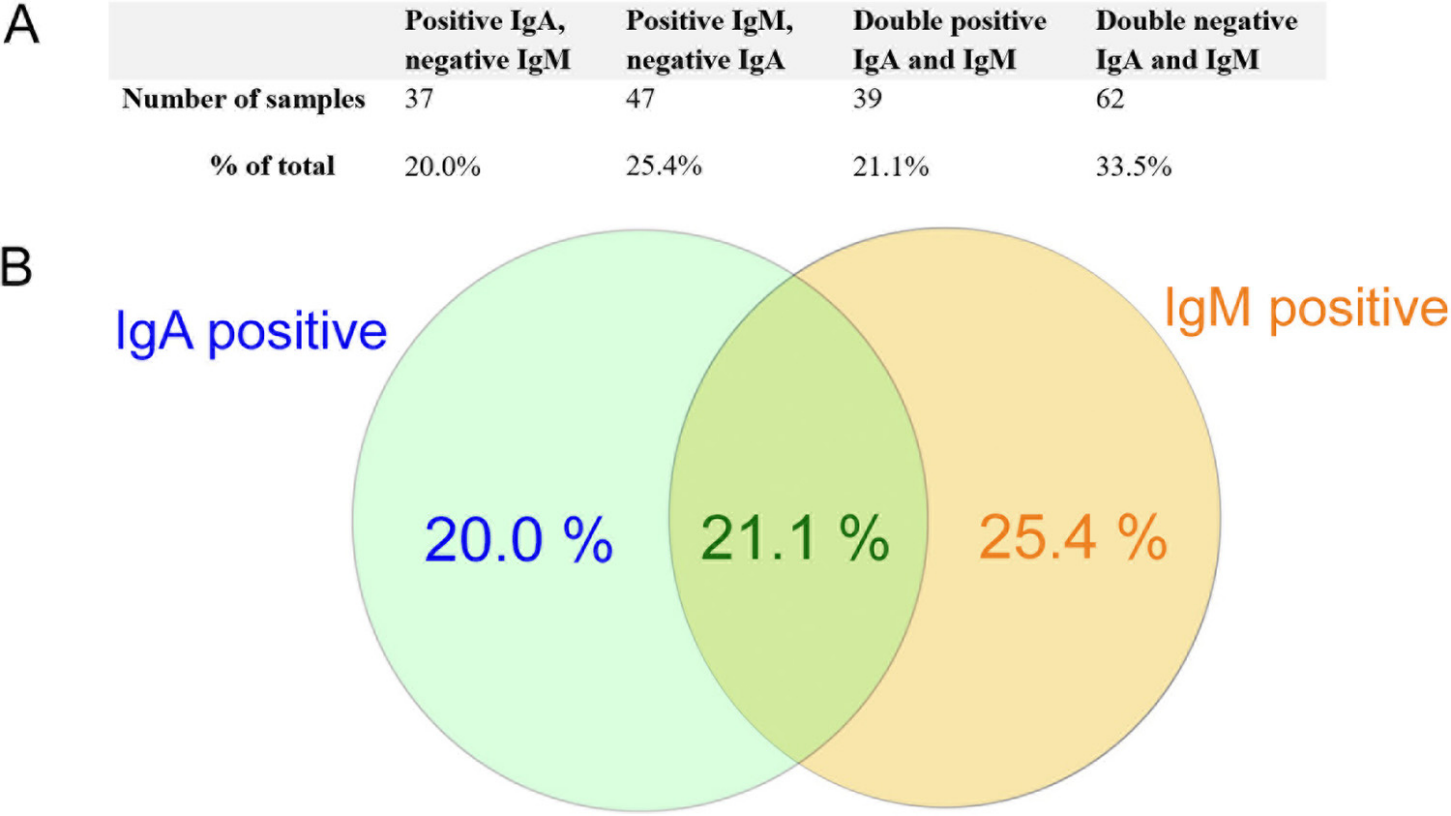
Analysis of blood serum samples via ELISA. (A) The results of specific IgA and IgM presence in the sera of patients. The number of positive samples and percentage of total are presented. (B) Venn diagram showing the percentage of total IgA-positive, IgM-positive, and double IgA/IgM-positive sera.

The patients’ demographic data and results of serum IgA and IgM evaluation are presented in Table 1. Working dilutions of the serum samples were 1:20. The obtained data suggest that both IgA and IgM were present in patients with ARF of all age groups. The analysis of ARF serum samples revealed 76 IgA-positive samples (41.1%), 86 IgM-positive samples (46.5%), and 39 double IgA/IgM-positive samples (21.1%). The obtained data suggest that the determination of IgM alone allows for serological confirmation of diagnosis in only 46.5% of cases; however, the determination of both IgM and IgA increased this rate up to 66.5% (Fig. 3). In the two control groups, both IgA and IgM antibodies were absent.

**TABLE 1:** Patient demographic data and results of IgA and IgM evaluation in sera

Demographic or disease characteristic	Total, *n*	IgM positive		IgA positive	
		*n*	%	*n*	%
Patients with ARF (*n* = 185)					
Sex					
Female	83	45	54.2	32	38.6
Male	97	39	40.2	40	41.2
Unknown	5	2	40.0	4	80.0
Age, yr					
<3	3	3	100.0	1	33.3
3–18	38	27	71.1	15	39.5
18–44	33	18	54.5	11	33.3
45–59	44	15	34.1	14	31.8
60–74	58	20	34.5	30	51.7
75–89	8	3	37.5	4	50.0
Unknown	1	0		1	100.0
Fever duration, days					
<3	50	23	46.0	14	28.0
4–6	72	34	47.2	29	40.3
7–16	59	28	47.5	30	50.8
Unknown	4	1	25.0	3	75.0

Q-fever-seropositive patients (*n* = 13)					
Sex					
Female	4	0		0	
Male	9	0		0	
Age, yr					
3–18	2	0		0	
18–44	6	0		0	
45–59	2	0		0	
60–74	2	0		0	
75–89	1	0		0	
Fever duration, days					
4–6	4	0		0	
7–16	7	0		0	
Unknown	2	0		0	

Healthy persons (*n* = 18)					
Sex					
Female	6	0		0	
Male	12	0		0	
Age, yr					
<3	1	0		0	
3–18	1	0		0	
18–44	8	0		0	
45–59	4	0		0	
60–74	2	0		0	
75–89	2	0		0	

**FIG 4:**
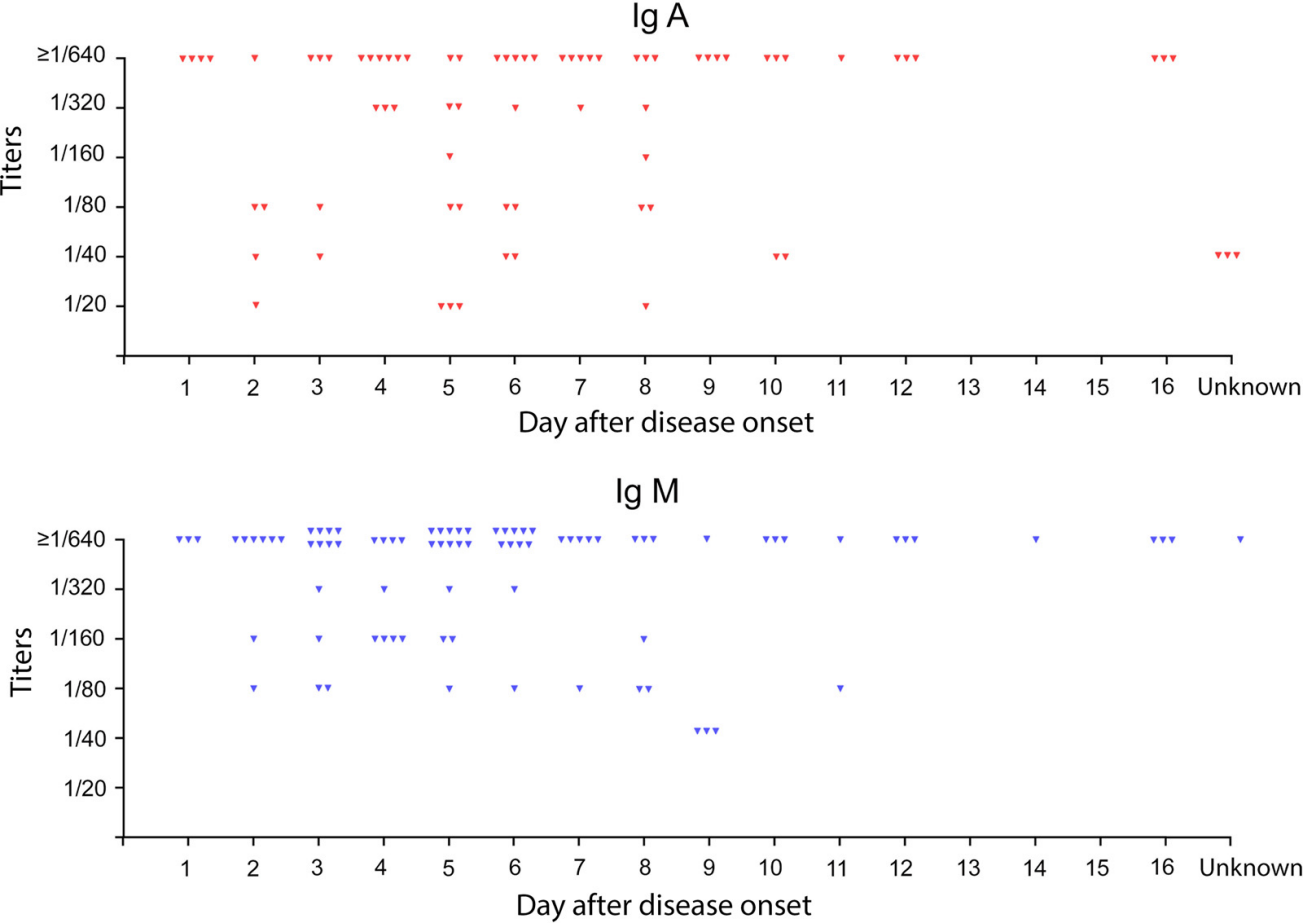
The titer and the number of positive samples according to the day after disease onset. Each positive serum sample is depicted as a dot.

After the first round of screening, the positive blood samples were diluted up to 1:640. The titer and the number of IgA- and IgM-positive samples according to the day from the onset of disease are shown in Fig. 4. The more detailed information regarding the number and the titer of IgA-positive/IgM-negative, IgA-negative/IgM-positive, double IgA/IgM-positive, and double IgA/IgM-negative sera according to the day from the onset of disease is presented in Table 2. The obtained data showed that 37 patients (20%) had relatively high serum levels of specific IgA antibodies (more than 1/640) and 62 patients (33.5%) had relatively high serum levels of specific IgM antibodies (more than 1/640). Interestingly, both IgA and IgM were detected at different time points from disease onset, from the 1st to 16th days.

**TABLE 2:** Evaluation of IgA and IgM titers in patients’ sera^*a*^

Day after disease onset	*n*	No. IgA pos, IgM neg (titer[s])	No. IgM pos, IgA neg (titer[s])	No. IgA pos, IgM pos (titer[s] of IgA and IgM)	No. IgA neg, IgM neg
0	1				1
1	6	3 (1/640, 1/640, 1/640)	2 (1/640, 1/640)	1 (1/640 and 1/640)	
2	17	1 (1/80)	4 (1/640, 1/640, 1/160, 1/80)	4 (1/640 and 1/640, 1/40 and 1/640, 1/80 and 1/640, 1/20 and 1/640)	8
3	26	4 (1/640, 1/640, 1/40, 1/80)	11 (1/640, 1/640, 1/640, 1/640, 1/640, 1/640, 1/640, 1/640, 1/320, 1/160, 1/80)	1 (1/640 and 1/80)	10
4	19	6 (1/640, 1/640, 1/640, 1/320, 1/320, 1/320)	6 (1/640, 1/640, 1/160, 1/160, 1/160, 1/320)	3 (1/640 and 1/640, 1/640 and 1/160, 1/640 and 1/640)	4
5	28	5 (1/20, 1/20, 1/20, 1/320, 1/320)	9 (1/640, 1/640, 1/640, 1/640, 1/640, 1/640, 1/640, 1/160, 1/80)	5 (1/80 and 1/320, 1/640 and 1/640, 1/160 and 1/640, 1/80 and 1/160, 1/640 and 1/640)	9
6	25	4 (1/640, 1/640, 1/40, 1/80)	5 (1/640, 1/640, 1/640, 1/640, 1/640)	6 (1/640 and 1/320, 1/640 and 1/640, 1/640 and 1/80, 1/320 and 1/640, 1/40 and 1/640, 1/80 and 1/640)	10
7	17	1 (1/640)	1 (1/80)	5 (1/640 and 1/640, 1/640 and 1/640, 1/640 and 1/640, 1/640 and 1/640, 1/320 and 1/640)	10
8	16	6 (1/640, 1/640, 1/320, 1/160, 1/80, 1/20)	4 (1/640, 1/160, 1/80, 1/80)	2 (1/640 and 1/640, 1/80 and 1/640)	4
9	7	1 (1/640)	1 (1/640)	3 (1/640 and 1/40, 1/640 and 1/40, 1/640 and 1/40)	2
10	9	3 (1/40, 1/40, 1/640)	1 (1/640)	2 (1/640 and 1/640, 1/640 and 1/640)	3
11	3	1 (1/640)	2 (1/80, 1/640)		
12	3			3 (1/640 and 1/640, 1/640 and 1/640, 1/640 and 1/640)	
13					
14	1		1 (1/640)		
15					
16	3			3 (1/640 and 1/640, 1/640 and 1/640, 1/640 and 1/640)	

a pos, positive; neg, negative.

In addition to IgA and IgM evaluation, IgG presence was studied in the sera. Obtained data and a Venn diagram showing the percentage of total IgA-positive, IgM-positive, IgG-positive, and double IgA/IgM, IgA/IgG, IgG/IgM-positive sera, as well as IgA/IgM/IgG-positive sera, are presented in Fig. S2.

We determined via Western blotting that both specific IgA and IgM antibodies from ARF patients’ sera recognize proteins of Rickettsia conorii (Fig. 2). To determine the ability of the specific IgA and IgM antibodies to recognize Rickettsia conorii lipopolysaccharides, we isolated LPS from the bacteria using a previously reported boiling method (13). We also isolated an LPS-free protein fraction from chromatographically purified antigen using a previously described method (14). Then, we quantified proteins and lipopolysaccharides in the chromatographically purified antigen, isolated LPS and chromatographically purified antigen, and determined that while chromatographically purified antigen contained both proteins and lipopolysaccharides, protein content in isolated LPS and LPS content in protein fraction were below the limit of detection. Then, we evaluated the antigenic ability of all three preparations via ELISA using eight positive sera (Table 3). We determined that IgM antibodies recognized both LPS and proteins. While IgA antibodies from all tested sera recognized proteins, only four sera from eight were positive in reaction with LPS (Table 3).

**TABLE 3:** Quantification of LPS and protein content and evaluation of antigenic activity in chromatography-purified LPS and protein fraction and in isolated LPS

Antigen	Quantity per mL (measured)		Quantity per well antigen dilution (calculated)		Titer	
	LPS, EU^*a*^	Protein, μg	LPS, EU	Protein, μg	IgA	IgM
Chromatographically purified LPS	7.3 × 10^4^	340 ± 14	2.1 × 10^2^	1	8 sera, 1/640	8 sera, 1/640
Protein fraction	<5	138 ± 5	<0.04	1	8 sera, 1/640	8 sera, 1/640
LPS	2.3 × 10^5^	<15	2.1 × 10^2^	<0.014	4 sera, 1/640; 4 sera, negative	8 sera, 1/640

a EU, endotoxin units.

Taken together, these data reveal the presence of specific IgA against causative agents in the blood of patients with ARF and indicate the clinical significance of IgA detection in patients with suspected ARF starting from the first days after disease onset.

## DISCUSSION

In Russia, approximately 200 to 300 cases of ARF are registered every year. In accordance with the data of the Rospotrebnadzor, the Russian Federal Service for Surveillance on Consumer Rights Protection and Human Wellbeing (data available at https://www.rospotrebnadzor.ru/activities/statistical-materials/), 295 cases of ARF in the Russian Federation were registered in 2014, with 314 in 2015, 299 in 2016, 176 in 2017, and 290 in 2018 (data for 2019 are not yet available). We used serum from 185 patients with ARF collected in the Astrakhan region, Russia, in 2019. As ARF is endemic to the Astrakhan region, it can be argued that the sera of the majority of patients with ARF who sought medical help in 2019 were included in this study.

In our analysis using Western blotting and ELISA, we showed that (i) specific IgA antibodies against causative agents of ARF are present in the blood from the early stage of disease and (ii) IgA evaluation has an important diagnostic value, with detection of both IgA and IgM allowing for a significant increase in the number of serologically confirmed cases.

Although we studied only blood from patients with ARF, it is highly likely that similar patterns of immunoglobulin secretion can be observed with another tick-borne spotted fever, Mediterranean spotted fever (MSF). Rickettsia conorii, the causative agent of ARF, is also responsible for MSF in several parts of Europe, Africa, and Asia (12). Moreover, taking into account the antigen similarity between spotted fever group *Rickettsia* species, the similarity of the clinical symptoms of fevers caused by them, and the cross-reactivity between secreted immunoglobulins to different rickettsias of this group (15–17), it may be hypothesized that detection of specific IgA in addition to IgM has an important diagnostic value for other tick-borne spotted fever rickettsioses distributed worldwide. However, this issue requires further investigation.

Previously published studies have revealed that specific IgM and IgG both predominantly interact with surface rickettsial proteins of high molecular masses, such as OmpA and OmpB (18–21). Our results indicate that proteins with similar molecular masses are predominantly recognized by IgA. It may be hypothesized that IgA, as well as immunoglobulins of other isotypes (M and G), recognizes the same immunogenic rickettsial proteins. This should be addressed in future studies.

Detection of IgA is used for the diagnosis of diseases caused by intracellular parasites, which predominantly infect via mucous membranes, such as many viruses, the protozoan Toxoplasma gondii, the bacterium Mycobacterium tuberculosis, and the obligate intracellular bacterium Chlamydia trachomatis (22–33). Here, we show that Rickettsia conorii, an obligate intracellular parasite, can induce predominant secretion of IgA in some cases. In fact, IgA was detected in 20.0% of the tested sera, but IgM was not. Further research is needed to determine the exact mechanism involved. Nevertheless, as IgA associates with mucous membranes, it can be assumed that at least in some cases, infection occurs through mucous membranes. Moreover, some rickettsias may be transmitted to humans via infected fluids (for example, ectoparasite feces) inoculated into damaged skin or mucous membranes, and this is the main route of transmission for typhus group *Rickettsia* (34). Although *Rickettsia* SFGs are considered to be transmitted only by tick feeding, cases of tick-borne spotted fever in a patient who denies tick attachment have been described (7). Thus, another mechanism of SFG *Rickettsia* may exist; it may be supposed that when crushing or removing a tick, people can transfer its fluids to their mucous membranes, causing rickettsia infection.

The usefulness of human IgG sorbent to prevent false-negative results due to the excess of IgG antibodies, and therefore to increase sensitivity of IgA and IgM detection, was reported previously. So, IgG sorbent was used in enzyme-linked immunofluorescence assay for detection of antibodies against Legionella pneumophila, Brucella, and Rickettsia conorii (35, 36). However, in other studies human IgG sorbent was not used for evaluation of specific IgM antibodies against *Rickettsia parkeri*, Rickettsia australis, Rickettsia conorii, Rickettsia rickettsii, Rickettsia typhi, and Orientia tsutsugamushi (37–39). So, the determination whether the use of human IgG sorbent can significantly increase sensitivity of IgA antibodies against Rickettsia conorii should be addressed in future studies.

Taken together, our data reveal an important diagnostic value of IgA evaluation for ARF. Therefore, to enhance the accuracy of diagnostics of tick-borne spotted fever rickettsiosis, both IgA and IgM should be analyzed in the serum samples of patients.

## MATERIALS AND METHODS

### Serum samples.

This was a retrospective anonymous study comprising 185 serum samples from patients diagnosed with ARF from the Astrakhan region, Russia. The serum was taken within May to October 2019 for routine diagnostic investigations for ARF and Q fever. Samples were stored at −80°C for quality control and performance evaluation properties not requiring ethical approval.

### Preparation of antigen for ELISA.

R. conorii was propagated in embryonated eggs, and formalin-inactivated and lyophilized bacteria served as a starting material. The contents of ampoules were resuspended in a buffer solution, 10 mM Tris-HCl, pH 7.5, 0.5 M sodium chloride (all from Sigma-Aldrich, USA), 10% 2-propanol (LiChrosolv from Merck, Germany). The suspension was sonicated using a Soniprep 150 Plus ultrasonic disintegrator (MSE, UK), with 6 rounds of sonication for 30 s at a frequency of 23 kHz with a maximal amplitude, with cooling on ice between rounds of sonication. During sonication, the probe of the ultrasound disintegrator was immersed in a glass of water, where the test tube with the treated antigen suspension was placed. The obtained suspension was centrifuged at 10,000 × *g* for 10 min at a temperature of 4°C. Size exclusion chromatography was performed using a chromatograph Äkta Explorer 10 system equipped with a spectrophotometer detector and a Superdex 200 prep-grade column (10 by 300 mm; GE Healthcare Bio-Science, Uppsala, Sweden) at a temperature of 4°C, with a running buffer consisting of 10 mM Tris-HCl, 0.5 M sodium chloride, 10% isopropanol, pH 7.5. Detection was performed at 280 nm.

### Preparation of protein fraction from antigen.

The removal of LPS from the antigen was performed according to a procedure described earlier with some modifications (14). The chromatographically purified LPS cooled at 4°C was diluted with working buffer (50 mM Tris-HCl, pH 7.5, 0.5 M NaCl, 4% Triton X-114, all from Sigma–Aldrich, USA) in a 1:1 (vol/vol) ratio. The obtained solution first was incubated with slight stirring at 4°C for 15 min, and then the solution was incubated at 37°C for 5 min. Then, the solution was centrifuged at 2,000 × *g* and at a temperature of 37°C for 5 min, and the resulting upper layer containing LPS-free protein fraction was collected. The procedure was repeated twice.

### Extraction of rickettsial LPS.

R. conorii was propagated in embryonated eggs, and formalin-inactivated and lyophilized bacteria served as a starting material. The lyophilized bacteria were suspended in solution containing 10 mM Tris-HCl, pH 7.5, 2 mM MgCl_2_ (all from Sigma-Aldrich, USA) and then sonicated using a Soniprep 150 Plus ultrasonic disintegrator (MSE, UK), for 1 min at a frequency of 23 kHz with a maximal amplitude. The LPS extraction was performed according to a previously described method with minor modifications (13). The obtained sonicated suspension was incubated at 95°C for 15 min with intensive vortexing every 5 min. Then, the suspension was centrifuged (10,000 × *g*, 10 min, 4°C), and supernatant was treated at 37°C firstly with Benzonase at a concentration of 2,500 U/mL (Merck, USA) for 2 hours and than proteinase K (Sigma-Aldrich, USA) at a concentration of 0.2 mg/mL overnight. Then, the solution was incubated for 15 min at 95°C and centrifuged (10,000 × *g*, 10 min, 4°C). The resulting supernatant was dialyzed against distilled water.

### Protein assay of antigens.

Proteins in soluble analytes were quantified using a Bradford method (40) reagent (Sigma, St. Louis, MO, USA), and the standard curve was obtained by plotting the optical density (OD) against known protein concentrations ranging from 0.25 to 1.0 mg/mL bovine serum albumin (BSA; Sigma, St. Louis, MO, USA) in phosphate-buffered saline (PBS).

### LAL test.

Bacterial lipopolysaccharide (endotoxin) quantification was determined using the *Limulus* amebocyte lysate (LAL) assay (catalog no. K50-643 J; Lonza Inc., Basel, Switzerland) according to the manufacturer’s instructions. Experiments and data analysis were performed using an ELx808 microplate reader (BioTek, Winooski, VT, USA) equipped with WinKQCL version 5.3.3 software (Walkersville, MD, USA).

### SDS-PAGE and Western blot analysis.

Serum samples were pooled from five samples and centrifuged (4°C, 10 min, 3,000 × *g*). Formalin-inactivated and lyophilized Rickettsia conorii bacteria were resuspended in isotonic phosphate-buffered saline (Sigma-Aldrich, USA), treated with ultrasound using a Branson S-450D instrument (Branson, USA), separated via SDS-PAGE, and stained using EZblue gel stain reagent (Sigma-Aldrich, USA) or transferred onto nitrocellulose membranes (GE Healthcare Life Science, USA). The membranes were probed with serum samples (1:200), and proteins of interest were detected with a horseradish peroxidase (HRP)-conjugated goat anti-human IgA antibody (1:2,000; Imtek, Russia) or goat anti-human IgM antibody (1:4,000; Imtek, Russia) and visualized using the Optiblot enhanced chemiluminescence (ECL) detection kit (ab133406; Abcam, UK) according to the provided protocol. Protein markers (ab115832; Abcam, UK) were used in experiments to estimate the molecular masses of proteins.

### ELISA.

A designed and validated N. F. Gamaleya National Research Center for Epidemiology and Microbiology ELISA kit was used for analysis. The developed ELISA was validated according to International Council for Harmonisation of Technical Requirements for Pharmaceuticals for Human Use (ICH) guidelines. The ELISA was shown to be linear within the analytical range and specific for diagnosis of spotted rickettsial fever using sera from patients with brucellosis, chlamydiosis, Q-fever, and bartonellosis. Robustness and the precision of the assay were verified in the Laboratory of Zooanthroponosis in the Pasteur Institute of Epidemiology and Microbiology, Saint-Petersburg, Russia. The developed assay sensitivity and precision adequately fulfilled all validation criteria as defined in the ICH guidelines.

The chromatographically purified antigen was diluted with 0.05 M carbonate buffer (pH 9.6) to a protein concentration of 10 μg/mL, pipetted into 96-well ELISA plates (C96 MaxiSorp Nunc-Immuno plate; Thermo Fisher Scientific, Denmark), and incubated at room temperature overnight. The buffer was prepared using sodium bicarbonate and sodium carbonate (Sigma-Aldrich, USA). To prevent nonspecific binding, 0.05 M carbonate buffer (pH 9.6) with the addition of 1% casein sodium salt was used (casein sodium salt from bovine milk; Sigma-Aldrich, USA).

Serum samples were diluted 1:20 for immunoglobulin detection. A 1:20 dilution for *Rickettsia*-specific IgM and IgG assay is widely used, and the dilution (1:20) is recommended as a diagnostic titer in a commercially available kit (Rickettsia conorii ELISA IgG/IgM kit; Vircell, Spain) (11, 41). Positive samples were then diluted up to 1:640. The samples were incubated with constant shaking. Specific conjugates (peroxidase-goat antibodies to human IgA, IgM, or IgG [Imtek, Russia]) were added to the antigen-antibody complex. A chromogen solution of 3,3′,5,5′-tetramethylbenzidine (TMB; Immunotech, Russia) was added for visualization of the reaction, which was stopped by the addition of 1 N sulfuric acid. The intensity of fluorescence measured as the optical density at 450 nm (comparison wavelength of 620 to 650 nm) was evaluated using a Stat Fax 4200 spectrophotometer (Awareness Technology, USA).

### Statistical analysis.

Data were analyzed using Microsoft Excel 2016 software (Microsoft Corp., Redmond, WA, USA) or GraphPad Prism 4.03 software (GraphPad Software, Inc., USA).
