# Supplementary material for: Diagnostic Value of IgA Antibody Measurement in Tick-Borne Spotted Fever (Astrakhan Rickettsial Fever)
Source: Microbiol Spectr. 2022 Apr 25;10(3):e01687-21. doi: 10.1128/spectrum.01687-21 (PMC9241626; doi:10.1128/spectrum.01687-21)
Supplement: SUPPLEMENTAL FILE 1 — Fig. S1 and S2. Download spectrum.01687-21-s001.pdf, PDF file, 0.9 MB [file spectrum.01687-21-s001.pdf]

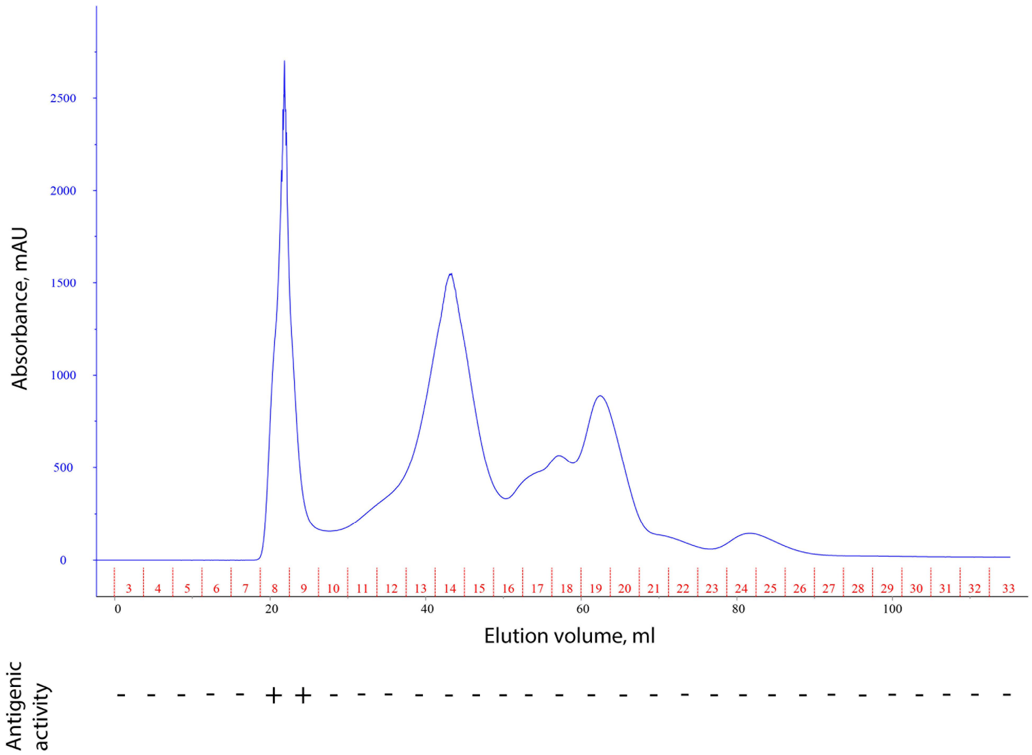


**Supplementary Fig. 1.** Purification of Rickettsia conorii antigen and evaluation its antigenic activity. A representative chromatogram of the purification process is presented. Each fraction was diluted 1:20 and then tested for antigenic activity via ELISA using ten combined positive sera.

+ means presence of antigenic activity, – means absence of antigenic activity.

A

| | Positive IgM | Positive IgA | Positive IgA, IgM | Positive IgA, IgM, IgG | Positive IgA, IgG | Positive IgM, IgG | Positive IgG | Negative |
|---|---|---|---|---|---|---|---|---|
| **Samples** | 23<br>12.4% | 17<br>9.2% | 10<br>5.4% | 29<br>15.7% | 20<br>10.8% | 24<br>13% | 24<br>13% | 38<br>20.5% |

B

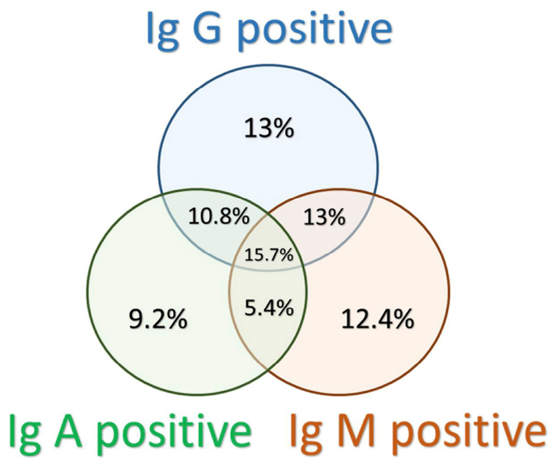


**Supplementary Fig. 2.** Analysis of blood serum samples via ELISA.
A. The results of specific IgA, IgM, IgG presence in the sera of patients. The number of positive samples and percentage of total are presented.
B. Venn diagram showing the percentage of total IgA-positive, IgM-positive, IgG-positive and double IgA/IgM, IgA/IgG, IgG/IgM-positive sera, as well as IgA/IgM/IgG positive sera
